# The relationship between the structure of firms’ human capital and corporate innovation performance

**DOI:** 10.1371/journal.pone.0321388

**Published:** 2025-04-08

**Authors:** Huimin Shao, Qing Jin, Yihang Guo, Fei Zhou, Walton Wider, Liangyan Lu

**Affiliations:** 1 College of Management, Yunnan Normal University, Kunming, China; 2 College of Economics, Yunnan Normal University, Kunming, China; 3 Faculty of Management, Shinawatra University, Pathum Thani, Thailand; 4 Faculty of Business and Communications, INTI International University, Nilai, Negeri Sembilan, Malaysia; 5 Department of Applied Economic Sciences, Wekerle Sandor Uzleti Foiskola, Budapest, Hungary; 6 Accounting and Finance Department, Yunnan College of Business Management, Kunming, Yunnan, China; Southwestern University of Finance and Economics, CHINA

## Abstract

Innovation in enterprise development is undoubtedly important. The relationship between its structure and the formation of enterprise innovation factors is at the core, so it is crucial to clarify them. Therefore, based on data on A-share listed companies in China from 2007 to 2022, this paper adopts a multilevel structural equation model to explore the effect of the structure of human capital on enterprise innovation performance. Unlike prior studies, this paper divides innovation performance by enterprises into “substantive innovation” and “strategic innovation.” Our results show that human capital plays an important role in promoting both strategic and substantive innovation, which is achieved mainly by increasing technological intensity, investing more in R&D, and receiving more government subsidies. In addition, the heterogeneity test shows that this facilitating role is significant in both samples, divided into the criteria of firm ownership and compensation incentives. However, they have different degrees of influence. This provides empirical support for the response by Chinese enterprises regarding the national development strategy and their new ideas about cultivating high-quality technological innovation and achieving sustainable development.

## 1. Introduction

Innovation is a crucial driver of economic growth and is essential for sustainable national economic development [1,2]. Because of the current international conditions, the optimal economic model has changed from the traditional one focused on rapid development to one based on high-quality development and from one based on factors to one motivated by innovation [3]. In this regard, the ability to innovate has become a key driving force in the ability of enterprises, as the main aspect of the country’s economic development, to sustain development and stand out. Data from the Ministry of Science and Technology (MOST) show that in 2021 enterprises led or participated in more than 680 (79%) of the 860 projects set up by the National Key R&D Program. Firms are at the center of R&D investment, project organization, and scientific and technological achievement, hence they play the main role in scientific and technological innovation. Therefore, enterprises need to develop high-quality innovations to maintain profitability and market competitiveness, thereby promoting sustainable enterprise development [4].

Innovation-driven development is based on talent, in which human capital plays a key role. Talent is the foundation of innovation; the drive for innovation is essentially a drive for talent, and human capital, which is at the center of scientific and technological innovation, is important in promoting greater capacity and enhancement in scientific and technological innovation [5,6]. Capital investment is also important, as is investment in human capital. High investment in human capital significantly increases enterprise innovation performance [7].

Therefore, the system for training human capital has been continuously optimized. In China, because of the expansion of colleges and universities, the quality and structure of overall human capital have undergone significant changes. The educated population, those with a bachelor’s degree or above, increased by 12.33% from 2001 to 2016. This ensures that firms will have sufficient talent to achieve innovative development if they increase their investment in human capital [8]. At the same time, to meet the demand for highly skilled human capital in national innovation, the state has also implemented supportive policies. The introduction in 2008 of a program to seek high-level talent overseas is important for accumulating human capital. Many high-level innovative and entrepreneurial talents and teams from other countries have been attracted by this program since its initiation. In addition, local governments are also constantly initiating programs to attract innovative talents and raise the level of regional innovation [3]. Comparable efforts are underway internationally. For example, the United Kingdom, as part of an effort to maintain its status as a global scientific and technological superpower, offers financial support to attract, train, and retain the world’s best researchers. This is achieved through the development and implementation of premier scholarship programs. Similarly, the German government has adopted a multifaceted approach to support steady talent development in international organizations. These efforts include providing information, career counseling, vocational training, and project funding, which highlights the importance of human resource development [9].

Overall, the progress enabled by science and technology innovation can effectively increase labor productivity at enterprises and improve their competitiveness. High-quality innovation is a main way to drive their sustainable development [10]. Therefore, the technological innovation ability of firms can greatly determine their survival, comparative advantage, market value, and return on investment [11]. Rational planning and allocation of human capital can accelerate the integration of internal scientific and technological innovation elements and raise organizational efficiency and the ability to achieve competitive advantages [12,13].

Human capital is the most important resource endowment, and it drives a country’s innovation development, technological progress, and high-quality economic development. The accumulation of human capital will increase the number of highly knowledgeable and skilled people, providing a more appropriate workforce for enterprise innovation. So, in the context of China’s implementation of the strategy of strengthening talent, whether human capital accumulation effectively promotes the innovation of China’s manufacturing enterprises to achieve the goal of high-quality development of the economy, especially in the context of the open economy, through what mechanism does human capital drive the innovation of manufacturing enterprises? The answer to this question not only helps to fully assess the important role of the structure of human capital in achieving an innovative performance of enterprises but also provides empirical evidence for realizing the high-quality development of China’s economy from the perspective of continuing to advance the reform of higher education and the transformation of human capital into an advanced form, as well as further promoting the opening of foreign trade.

However, numerous questions remain about the relationship between a firm’s human capital structure and corporate innovation performance. Can different configurations of human capital structure effectively promote improvement in corporate innovation performance? What is the underlying mechanism of this influence? Are the effects of different structures of human capital heterogeneous? Clarifying these issues has significant practical implications for enterprises concerning human resource investment decisions, allocation of technological innovation talents, and optimization of human capital structure. This paper explores the impact of the structure of human capital on corporate innovation performance and its mechanisms through empirical analysis, providing theoretical support for firms’ innovative development and coordinated economic and social development.

The paper makes some marginal contributions, as follows. First, concerning the research perspective, prior literature primarily discusses the economic impact of human capital at the macro level. In contrast, micro-level studies focus mainly on the causal effect of human capital on technological progress, enhancement of total factor productivity, and export upgrades. However, no consensus has been reached about whether these factors promote or inhibit innovative firm activities. This paper systematically examines how the structure of human capital affects firms’ innovation performance and reveals its underlying mechanisms from a micro-enterprise perspective. Second, regarding the research content and conclusions, this paper divides enterprises’ innovation performance into strategic and substantive innovation. It analyzes the impact of the structure of human capital on the innovation performance of enterprises from different perspectives to put forward policy recommendations and support in a more targeted manner. Third, in terms of the transmission mechanism, the paper analyzes the impact of the structure of human capital on firms’ innovation performance from the internal and external perspectives of enterprises and the heterogeneity of the impact from different perspectives so that we can offer appropriate recommendations.

## 2. Literature review and research hypotheses

### 2.1. Human capital structure and firm innovation performance

Schultz first articulated the concept of human capital as capital embodied in workers, such as their level of knowledge, skills, culture, and technology. Human capital, as “living capital,” is characterized by innovation and creativity [13]. The development of an enterprise encompasses the attraction and subsequent absorption of global professionals, which enhances the internal structure of its human capital. At the same time, it facilitates movement by personnel and technology among enterprises in different regions, driving technological innovation and economic development more broadly [14].

Many scholars have confirmed the positive effect of human capital on firms’ innovation performance in different types of research. Wright et al. [15] state that the rational allocation of human capital can effectively raise organizational innovation performance. Chen and Ding [16] find a positive relationship between the influence of the educational structure of human capital on total factor productivity and the business performance of enterprises from the perspective of the education level. Li and Cao [17] construct a dummy variable for the policy of enrollment expansion of the number of potential students who can take the college entrance examination as the time point of policy implementation; using the difference-in-difference method, they confirm that enhancement in human capital has a positive impact on the innovation performance of manufacturing enterprises in China and indicate that expansion of postgraduate education can lead to a superposition effect. Tong and Zhang [18] discuss the role of high-level human capital investment in promoting innovative output by Chinese export enterprises from two perspectives: increasing the types of new products and improving the quality of existing products. Therefore, we propose the following hypothesis:

*H1: Optimizing the structure of human capital at a firm can improve its innovation performance.*

### 2.2. Technological intensity, human capital structure, and firm innovation performance

Grossman et al. [19] believe that having more highly educated human capital is the main factor that affects innovation and increases in the highly skilled workforce benefit innovation. Papageorgiou [20] similarly holds that the effectiveness of innovation activities mainly depends on the investment in employees with secondary education or above. Chang et al. [21] find that employees at high professional and technical levels are usually multitaskers, making them more creative and conducive to enterprise innovation. For example, executives with experience as inventors can raise the intensity of enterprise capital investment, the number of patents granted, and the efficiency of R&D innovation [22].

The number of R&D staff at an enterprise, particularly the number of high-level R&D staff, represents technical strength, which is an important factor that affects the success of R&D projects and can ensure enterprise innovation. When the proportion of high-level talent is higher, enterprises’ innovation potential and performance are greater. These positions usually have higher requirements concerning employees’ professional mastery, capacity for learning and application, and active thinking. When they account for a higher share of an enterprise’s human capital, the firm has a stronger tendency to innovate and a higher level of creativity, which implies higher R&D efficiency and quality—this can effectively enhance the enterprise’s innovation performance. As Schultz’s [13] “internal effect” theory shows, the human capital formed by “formal and informal education” can improve labor productivity, decision-making efficiency, and output growth through the accumulation of knowledge and skills. This means that a firm’s high level of human resources is often indicative of whether it has a high level of R&D capability and production technology and thus the quality of the output from subsequent innovation performance. Based on this, we propose the following hypothesis:

*H2: Optimizing the structure of human capital at firms can improve innovation performance by increasing technological intensity.*

### 2.3. R&D investment, human capital structure, and firm innovation performance

According to the resource-based theory (RBV), a good structure of human capital is the basis for transforming knowledge, skills, and capital into market demand. The combination of higher human capital and physical capital can benefit enterprises through innovative activities. When an enterprise’s human capital structure is higher, production factors and R&D investment are needed, and innovation performance is higher [23]. By constructing a concept data model, Li et al. [24] find that the increase in innovation investment is conducive to transforming accumulated intellectual capital into innovation output, which has a significantly positive impact on enterprise innovation performance. Li et al. [25] use the back propagation neural network algorithm to analyze the influence of multifactor input on enterprise innovation performance. They state that R&D investment can significantly promote innovation performance at the stage of technological development but might inhibit enterprise innovation at the stage of achievement transformation. Yang et al. [26] reach a similar conclusion. They find a single threshold for R&D capital investment, which is significantly positively correlated with innovation performance indicators when it does not exceed the critical value; if it exceeds the critical value, it has a slightly inhibitory effect, but it is not apparent.

The investment of sufficient R&D funds can positively impact firms’ innovation performance: when the investment amount is high, an enterprise can produce a large amount of innovation output [27,28]. At the same time, large-scale R&D innovation can effectively withstand some unavoidable failure [29]. A good human capital structure can make full use of R&D resource inputs and can thereby help enterprises optimize the allocation of R&D resources and improve innovation performance. This means that employees with more professional knowledge, a deeper understanding of the industry, and cutting-edge technology can help enterprises achieve this optimal allocation, reduce ineffective and inefficient R&D investment, and thus improve innovation performance. Based on this, we propose the following hypothesis:

*H3: Optimizing a firm’s human capital structure can improve its innovation performance by increasing investment in R&D.*

### 2.4. Government subsidies, human capital structure, and firms’ innovation performance

Fiscal subsidies by local governments are essential for improving the quality of corporate innovation [30]. This subsidy to firms is in the form of direct financial allocations as well as indirect tax incentives. Both approaches can improve firms’ innovation performance [31]. Enterprises benefit not only from their own internal strengths but from maintaining abundant human resources, which is the main way in which the government subsidies operate.

Enterprises are helped by having good-quality human resources because of their influence on innovation. Therefore, the government designs preferential policies for enterprises that will help them attract highly qualified personnel and expand support for project applications and the allocation of funds [32]. Highly skilled human capital enables enterprises to externalize tacit knowledge and absorb explicit knowledge, thus improving their innovation efficiency [33]. As a policy tool, government subsidies aim to encourage enterprise innovation and development. Government subsidies can alleviate the financial pressure on R&D and attract talent by giving economic support to enterprises directly or indirectly through financing. In this way, enterprises obtain more funds for cultivating high-level talents and improving their human capital stock [34].

Countries all over the world offer government subsidies to help nurture talent.. Germany has rich and diverse projects for funding skills training, comprising ordinary citizens, talents in particular fields, and civil servants. Japanese programs for training workers are multilayered to meet the need for different skill levels and development stages, which reflects openness about skills building and the importance that the government places on talent development [35]. Based on this, we propose the following hypothesis.

*H4: Optimizing the human capital structure of enterprises can improve their innovation performance if they obtain higher government subsidies.*

**Fig 1:**
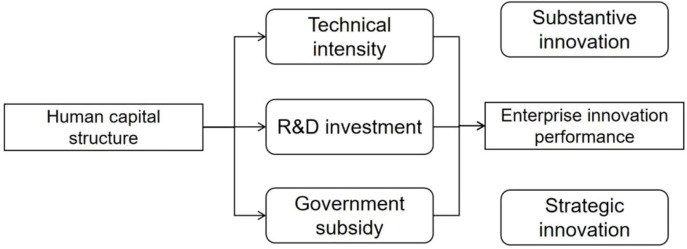
Theoretical framework.

Fig 1 shows the theoretical framework of this study.

### 2.5. Critical analysis

Our review of previous literature and research shows that current research on the relationship between the optimization of human capital structure and enterprise innovation performance has yielded rich and diverse results. Although research on human capital and enterprise performance has been carried out for a long time, no consensus has been reached. Early scholars believed that performance improvement is hindered by emphasizing only human capital investment but overlooking problems in investment efficiency; rather, raising the human capital level enables enterprises to achieve synergistic effects, creating two U-shaped relationships. Recent studies have shown that human capital can raise efficiency in resource interaction and transformation at an enterprise and has a driving effect on enterprise performance. The relevant literature mostly analyzes the relationship between them directly, starting with human capital value added, experience, motivation, and other aspects of intellectual capital. However, few studies have explored how human capital affects performance, so this needs to be investigated in depth.

Most studies have measured firms’ innovation performance in a single general research framework. However, few have focused on the differences between substantive and strategic innovation, which not only define the quality of a firm’s current innovation performance but also reflect the effectiveness of its human resource utilization and, thus, its ability to sustain innovation. Substantive innovation drives technological progress in high technology, whereas strategic innovation only relates to policy orientation and generally requires only minor, low-tech innovation.

Therefore, unlike prior papers, this study comprehensively incorporates substantive and strategic innovation into the scope of firms’ innovation performance, explores the relationship between human capital structure and a firm’s innovation performance from two perspectives, and further analyzes the role of mechanisms and influence paths for enriching the existing research. In addition, prior studies use a single measure of the structure of human capital, mainly focusing on the level of education and the number of R&D personnel, lacking an in-depth discussion on the diversity in the structure of human capital. The existing research on the role of government subsidies in the relationship between human capital and firms’ innovation performance is insufficiently detailed. It mostly focuses on the direct impact of subsidies on firm innovation while ignoring their indirect impact on human capital structure.

## 3. Research design

### 3.1. Data description

Based on the completeness and timeliness of the available data, we use a sample of information on all of China’s A-share listed companies from 2007 to 2022. The market data comes from the China Stock Market and Accounting Research (CSMAR) database and the national statistical yearbook (2007–2022), except for patent data on the innovation performance of enterprises, which is obtained from the China Research Data Service (CNRDS).

We use the following methods to ensure the stability and validity of the data and eliminate the influence of outliers. First, we omit samples from the financial and real estate industries to ensure data consistency because they use a different financial accounting system from the other sectors. Second, we omit samples for ST (special treatment) and ST * enterprises because, during the sample period, these enterprises had continuous losses and financial anomalies, unlike other enterprises. Third, we omit enterprises with incomplete data on the main variables to ensure data reliability. Fourth, we omit enterprises with an asset-liability ratio less than 0 or greater than 1 because that could indicate that they are insolvent or have abnormal operations, which might yield misleading results. Fifth, we omit enterprises with total assets of 0. We obtain a final sample of 40,093 valid observations.

### 3.2. Variable setting

#### 3.2.1. Explained variable.

Following Li et al. [36], we distinguish substantive innovation and strategic innovation by measuring the innovation performance of enterprises from two perspectives: InQuantity and InQuality. Because authorized patents, as an important achievement that reflects the innovation output of enterprises, are subject to supervision and inspection at the national level, have certain authority, and reflect the output of enterprise innovation activities, we use the number of patents as a proxy variable for innovation [37]. Patent authorization is subject to approval procedures, and this process from application to authorization takes time; moreover, the patent application requires certain costs and human resources, representing enterprises’ innovation consciousness. In order to measure the relationship between R&D input and innovation output in the current period more accurately, we use the number of patent applications instead of the number of patents granted.

Patents come in three types: invention patents, utility model patents, and design patents. Invention patents have high input costs, complex applications, and stricter technical requirements, reflecting innovation’s essence. However, the reason for enterprises to apply for utility models and design patents to obtain government subsidies, which is a strategic consideration. In summary, we use the number of non-invention patent applications by sample enterprises to reflect the number of innovations and the number of invention patent applications to measure the quality of innovation.

#### 3.2.2. Explanatory variables.

Human capital structure is measured by the number of employees with an undergraduate degree or above (lnEdu), as is commonly used in prior studies. The level of education determines the ability of employees to learn and absorb knowledge, which is the embodiment of an enterprise’s overall cultural quality, and highly educated employees pay more attention to enhancing their self-worth. As a result, the motivation for engaging in R&D is also stronger, which in turn helps improve enterprises’ innovation performance [38,39]. Additionally, considering the availability and openness of data, the data on the structure of human capital released by listed companies generally includes the proportion of employees with at least a bachelor’s degree. That is also why this data is commonly used in current research, so we reference prior studies and use this indicator to measure the structure of human capital.

#### 3.2.3. Control variables.

Following previous studies, we include the following control variables. The variables for firm characteristics include firm size (lnSize), profitability (Roa), solvency (Lev), cash flow (Cash), and listing age (lnAge), and the variables for corporate governance comprise property rights (State) and equity concentration (Top1). In addition, we control for other factors that might affect the innovation performance of enterprises, such as the market level (market) and regional economic strength (lnGDP) [36,40,41]. These control variables are widely employed

In research focused on firms, and we use them for two reasons. First, the data come from financial reports disclosed by enterprises, so they have a certain degree of authenticity and accessibility. Second, these financial indicators are typically used in management studies and can reflect the actual financial status of enterprises to a certain extent. All relevant variables and their meanings are presented in Table 1.

**Table 1:** Variable definitions.

Type	Variable	Variable Label	Definition
Explained variable	Innovation performance	InQuantity	In (number of utility model patents applied for by an enterprise in the current year + number of design patents + 1)
		InQuality	In (number of invention patents applied for by an enterprise in the current year + 1)
Explanatory variable	Human capital structure	lnEdu	In (number of employees with a bachelor’s degree or above + 1)
Mediating variables	Technology intensity	lnRDperson	In (number of R&D personnel in the enterprise + 1)
	Degree of investment in R&D	lnRD	In (R&D investment amount + 1)
	Degree of government support	lnGov	In (amount of government subsidy in the current period + 1)
Control variables	Enterprise size	lnSize	In (Total assets)
	Profitability	ROA	Net profit/total assets
	Solvency	Lev	Total liabilities/total assets
	Cash flow	Cash	Operating cash flow/total assets
	Listing age	lnAge	LN (IPO Age + 1)
	Type of firm ownership	State	1 for state-owned enterprises, 0 otherwise
	Equity concentration	Top1	Largest shareholding ratio of the largest shareholder
	Marketization level	Market	Market-oriented level of the province in which the enterprise is located
	Regional economic strength	lnGDP	ln(GDP of the province in which the company is located)

#### 3.2.4. Model settings.

This paper uses models 1 and 2 to test H1. InQuantity and InQuality on the left side of the equation reflect the innovation performance of the enterprise from the perspective of the quantity and quality of enterprise innovation, respectively. In contrast, lnEdu on the right side of the equation reflects the human capital of the enterprise. Controls are control variables, with i.id and i.year indicating that the individual and year-level effects are controlled for, respectively [42]. In addition, robust standard errors are used in the regression to enhance the validity of the estimates. If the pre-lnEdu coefficient is significantly positive, then, the human capital structure of the enterprise can drive improvement in the company’s innovation performance. That is, H1 is confirmed.


$${\text{InQuantity=α}}_{\text{0}}{\text{+α}}_{\text{1}}{\text{lnEdu+α}}_{\text{2}}\text{Controls+i}\text{.id+i}\text{.year+ε}$$ (1)

$${\text{InQuality=α}}_{\text{0}}{\text{+α}}_{\text{1}}{\text{lnEdu+α}}_{\text{2}}\text{Controls+i}\text{.id+i}\text{.year+ε}$$ (2)

H2, H3, and H4 are tested using the following mediating effect model.


$${\text{M=α}}_{\text{0}}{\text{+α}}_{\text{1}}{\text{lnEdu+α}}_{\text{2}}\text{Controls+i}\text{.id+i}\text{.year+ε}$$ (3)

$${\text{InQuantity=α}}_{\text{0}}{\text{+α}}_{\text{1}}{\text{M+α}}_{\text{2}}{\text{lnEdu+α}}_{\text{3}}\text{Controls+i}\text{.id+i}\text{.year+ε}$$ (4)

$${\text{InQuality=α}}_{\text{0}}{\text{+α}}_{\text{1}}{\text{M+α}}_{\text{2}}{\text{lnEdu+α}}_{\text{3}}\text{Controls+i}\text{.id+i}\text{.year+ε}$$ (5)

where M is a range of mediating variables, including the number of R&D personnel (lnRDperson), R&D funding (lnRD), and government grants (lnGov). If ${\text{α}}_{\text{1}}$ in Equation (3) is significant, then, the mediating variable affects the innovation performance of the enterprise, according to the three-step test method based on the mediating effect [43]. If ${\text{α}}_{\text{1}}$ in Equation (1) is significant, and ${\text{α}}_{\text{1}}$ and ${\text{α}}_{\text{2}}$ in Equation (4) are both significant, then a partial mediating effect exists, that is, the human capital of the enterprise affects the company’s innovation performance, and this impact on the innovation performance is transmitted by both human capital and mediating variables. If ${\text{α}}_{\text{1}}$ is significant in Equation (1), and ${\text{α}}_{\text{1}}$ is significant, but ${\text{α}}_{\text{2}}$ is not significant in Equation (4), then, a complete mediating effect exists, and the influence of an enterprise’s human capital on innovation performance is completely transmitted through this mediating variable. Equations (2) and (5) are the same as Equations (1) and (4).

## 4. Empirical results

### 4.1. Descriptive statistics

Table 2 lists the descriptive statistics for the variables of interest and the correlation coefficients of each variable with firms’ innovation performance. The important variable in the research sample, the mean of InQuantity is 2.153, the minimum is 0, and the maximum is 9.088. The mean value of InQuality is 1.923, the minimum is 0, and the maximum is 9.087, indicating large differences in innovation performance among enterprises. The other variables are consistent with those in prior studies.

**Table 2:** Descriptive statistics.

Variable	Observations	Mean	Standard deviation	Minimum	Median	Maximum
InQuantity	40,093	2.153	1.715	0	2.1972	9.088
InQuality	40,093	1.923	1.598	0	1.7918	9.087
lnEdu	40,093	5.574	2.139	0	5.8201	12.78
lnSize	40,093	22.07	1.319	13.08	21.8755	28.64
ROA	40,093	0.041	0.170	−3.164	0.0401	22.01
Lev	40,093	0.412	0.206	0.0017	0.4038	0.999
Cash	40,093	0.0488	0.100	−11.06	0.0482	2.222
lnAge	40,093	1.973	0.952	0	2.1972	3.497
State	40,093	0.386	0.487	0	0	1
Top1	40,093	0.345	0.150	0.0029	0.3228	0.900
Market	40,093	7.372	1.579	−0.230	7.52	11.71
lnGDP	40,093	10.48	0.850	5.841	10.5704	11.77

### 4.2. Baseline statistics

Models 1 and 2 preliminarily confirm H1; the results are shown in Table 3. The first two columns are the bidirectional fixed-effect model results with InQuantity as the dependent variable. In column 1, the coefficient of human capital is significantly positive, indicating that optimization of the human capital structure of enterprises can increase the quantity of their innovation output. In column 2, the coefficient for human capital remains significantly positive after adding the control variable, confirming our prior findings. Columns 3 and 4 change the dependent variable from InQuantity to InQuality, demonstrating the relationship between the structure of an enterprise’s human capital and the quality of innovation output. The results show that the number of employees with at least a bachelor’s degree significantly positively affects the quality of innovation output, regardless of whether control variables are added. High-quality employees tend to have more affluent and diversified knowledge and skills, enabling them to achieve greater technological iteration and innovation, manage and use the enterprise’s resources, promote continuous innovation and change, and respond better to fierce market competition. Therefore, we find that optimizing the structure of human capital improves both the quantity and the quality of innovation output; that is, it can improve companies’ innovation performance; H1 is confirmed.

**Table 3:** Baseline regression.

	(1)	(2)	(3)	(4)
	InQuantity	InQuantity	InQuality	InQuality
lnEdu	0.110***	0.048***	0.118***	0.053***
	[24.24]	[10.21]	[28.41]	[12.41]
lnSize		0.401***		0.430***
		[37.45]		[44.10]
ROA		0.061**		0.053**
		[2.23]		[2.15]
Lev		0.002		−0.050
		[0.05]		[−1.24]
Cash		−0.126**		−0.066
		[−2.53]		[−1.45]
lnAge		0.045***		0.035***
		[3.11]		[2.60]
State		0.000		0.000
		[0.00]		[0.00]
Top1		−0.009		−0.249***
		[−0.12]		[−3.75]
Market		0.001		−0.014 *
		[0.06]		[−1.67]
lnGDP		0.156**		0.367***
		[1.97]		[5.08]
Year effect	Yes	Yes	Yes	Yes
Individual effects	Yes	Yes	Yes	Yes
_cons	0.828***	−9.123***	0.656***	−11.643***
	[31.65]	[−11.91]	[27.31]	[−16.70]
*N*	40,093	40,093	40,093	40,093
*R* ^2^	0.281	0.312	0.297	0.338

* , **, and *** show statistical significance at 10%, 5%, and 1% levels, respectively.

### 4.3. Mediating effect regression

To confirm H2, H3, and H4, we perform the following mechanism tests, and the results are shown in Tables 4 and 5. The first two columns in the tables show the mediating effect of technology intensity on human capital and innovation performance, represented by the number of R&D personnel. The first column replaces the dependent variable with the number of R&D personnel (lnRDperson), and the coefficient of lnEdu is 0.151, which is significantly positive at the 1% level, indicating that the optimization of the human capital structure of the enterprise can increase the number of R&D personnel. Column 2 adds the number of developers (lnRDperson) as an explanatory variable, and the explanatory variables are InQuantity and InQuality, respectively. The coefficients of lnRDperson are 0.062 and 0.065, respectively, which are significantly positive at the 1% level. The increase in the number of R&D personnel can provide technical support for enterprise innovation in terms of both the quantity and the quality of innovation output and improve the company’s innovation performance; H2 is confirmed. In addition, after lnRDperson is added, the coefficients of lnEdu are 0.039 and 0.043, respectively, lower than those for the benchmark regression (0.048 and 0.053) but still significantly positive. Based on the description of the coefficient of the mediating model, ${\text{α}}_{1}$ in model 1 is significant, and ${\text{α}}_{1}$ and ${\text{α}}_{2}$ in Equation (4) is both significant, indicating that technology intensity partially mediates the relationship between human capital and innovation performance. In other words, the influence of human capital optimization on enterprise innovation performance is partly achieved by raising the degree of technology intensity. In general, when an enterprise has more R&D personnel, it has greater scientific R&D capability and technological innovation capacity. A larger number of R&D personnel can absorb more wisdom, increase the utilization efficiency of equipment and technology, and more easily evoke enthusiasm by the team, thus increasing the efficiency of technological innovation and driving innovation performance by the enterprise.

**Table 4:** Mediating effects: InQuantity.

Variables	(1)	(2)	(3)	(4)	(5)	(6)
	Technology-intensive mediating effect		Mediating effect of R&D investment		Mediating test of the degree of government support	
	lnRDperson	InQuantity	lnRD	InQuantity	lnGov	InQuantity
lnEdu	0.151***	0.039***	0.239***	0.041***	0.122***	0.047***
	[31.05]	[8.15]	[12.17]	[8.72]	[8.39]	[9.95]
lnRDperson		0.062***				
		[11.96]				
lnRD				0.030***		
				[23.95]		
lnGov						0.009***
						[5.00]
lnSize	0.544***	0.368***	1.198***	0.365***	0.942***	0.393***
	[49.31]	[33.27]	[26.80]	[34.00]	[28.40]	[36.26]
ROA	−0.142***	0.069**	−0.272**	0.069**	−0.134	0.061**
	[−5.06]	[2.56]	[−2.40]	[2.55]	[−1.59]	[2.25]
Lev	0.088 *	−0.003	−1.064***	0.035	0.013	0.004
	[1.94]	[−0.07]	[−5.79]	[0.79]	[0.10]	[0.08]
Cash	0.234***	−0.140***	0.375 *	−0.137***	−0.127	−0.125**
	[4.59]	[−2.83]	[1.81]	[−2.78]	[−0.82]	[−2.52]
lnAge	0.087***	0.040***	−0.948***	0.074***	−0.555***	0.050***
	[5.80]	[2.75]	[−15.59]	[5.10]	[−12.29]	[3.43]
Top1	−0.124 *	−0.001	−0.603**	0.009	0.737***	−0.017
	[−1.65]	[−0.02]	[−1.98]	[0.13]	[3.26]	[−0.23]
Market	−0.017 *	0.002	−0.027	0.001	−0.029	0.001
	[−1.79]	[0.17]	[−0.73]	[0.15]	[−1.05]	[0.07]
lnGDP	0.185**	0.145 *	−0.795**	0.180**	−0.468 *	0.157**
	[2.27]	[1.83]	[−2.40]	[2.29]	[−1.90]	[1.98]
Year effect	Yes	Yes	Yes	Yes	Yes	Yes
Individual effects	Yes	Yes	Yes	Yes	Yes	Yes
_cons	−13.186***	−8.311***	−14.389**	−8.688***	−14.764***	−8.958***
	[−16.73]	[−10.83]	[−4.51]	[−11.43]	[−6.22]	[−11.69]
N	40093	40093	40093	40093	40081	40081
R-sq	0.585	0.315	0.540	0.323	0.541	0.313

* , **, and *** show statistical significance at 10%, 5%, and 1%, respectively.

**Table 5:** Mediating effects: InQuality.

	(1)	(2)	(3)	(4)	(5)	(6)
	Technology-intensive mediating effect		Mediating effect of R&D investment		Intermediary test of the degree of government support	
	lnRDperson	InQuality	lnRD	InQuality	lnGov	InQuality
lnEdu	0.151***	0.043***	0.239***	0.046***	0.122***	0.052***
	[31.05]	[10.00]	[12.17]	[10.81]	[8.39]	[12.12]
lnRDperson		0.065***				
		[13.97]				
lnRD				0.030***		
				[26.21]		
lnGov						0.009***
						[5.71]
lnSize	0.544***	0.395***	1.198***	0.394***	0.942***	0.421***
	[49.31]	[39.24]	[26.80]	[40.38]	[28.40]	[42.74]
ROA	−0.142***	0.063**	−0.272**	0.062**	−0.134	0.054**
	[−5.06]	[2.53]	[−2.40]	[2.51]	[−1.59]	[2.19]
Lev	0.088 *	−0.055	−1.064***	−0.018	0.013	−0.049
	[1.94]	[−1.39]	[−5.79]	[−0.45]	[0.10]	[−1.23]
Cash	0.234***	−0.081 *	0.375 *	−0.077 *	−0.127	−0.065
	[4.59]	[−1.80]	[1.81]	[−1.72]	[−0.82]	[−1.44]
lnAge	0.087***	0.029**	−0.948***	0.063***	−0.555***	0.039***
	[5.80]	[2.18]	[−15.59]	[4.78]	[−12.29]	[2.96]
Top1	−0.124 *	−0.241***	−0.603**	−0.231***	0.737***	−0.257***
	[−1.65]	[−3.63]	[−1.98]	[−3.51]	[3.26]	[−3.86]
Market	−0.017 *	−0.013	−0.027	−0.013	−0.029	−0.013
	[−1.79]	[−1.54]	[−0.73]	[−1.59]	[−1.05]	[−1.62]
lnGDP	0.185**	0.355***	−0.795**	0.391***	−0.468 *	0.368***
	[2.27]	[4.92]	[−2.40]	[5.46]	[−1.90]	[5.09]
Year effect	Yes	Yes	Yes	Yes	Yes	Yes
Individual effects	Yes	Yes	Yes	Yes	Yes	Yes
_cons	−13.186***	−10.780***	−14.389***	−11.210***	−14.764***	−11.478***
	[−16.73]	[−15.45]	[−4.51]	[−16.23]	[−6.22]	[−16.45]
N	40093	40093	40093	40093	40081	40081
R-sq	0.585	0.342	0.540	0.351	0.541	0.339

* , **, and *** show statistical significance at 10%, 5%, and 1%, respectively.

Similarly, columns 3 and 4 in Tables 4 and 5 confirm H3. Adequate capital investment in R&D means that enterprises have more funds for supporting S&T innovation activities, including purchasing advanced R&D equipment, constructing laboratories, and adding high-quality R&D talents. It also means that enterprises with more capital investment in R&D have more opportunities for exploring new scientific and technological fields, driving the development of new products, and upgrading the level of technology, thus accelerating innovation and improving innovation performance.

The results in columns 5 and 6 in Tables 4 and 5 confirm H4. Government subsidies can effectively mitigate financial pressure on enterprises and encourage them to engage in technological innovation in key areas. This not only stimulates innovation by enterprises but also enhances their R&D environment. Government subsidies can also provide enterprises with R&D cooperation opportunities and technical support, facilitating innovations’ transformation and application. In this way, innovations by enterprises can be transformed into actual productivity more quickly and have economic benefits for enterprises.

Thus, regardless of the quantity or quality of innovations, human capital optimization partly affects firms’ innovation performance through technological intensity, R&D investment, and government subsidies. H2, H3, and H4 are confirmed.

### 4.4. Heterogeneity testing

To investigate the influence of sample heterogeneity on the results, following Zhang et al. [44], we divide the samples into groups based on the firm’s ownership (vs. non–state owned) and compensation incentives and then compare the results across the groups. Table 6 reports the results of heterogeneity based on firm ownership, showing that, regardless of whether firms are state owned or non–state owned, the quantity and quality of innovation output are positively affected by human capital. The influence of human capital on the quantity of innovation output is higher at state-owned enterprises (SOEs) than at non-SOEs in terms of value and significance—that is, SOEs put more emphasis on strategic innovation.

**Table 6:** Heterogeneity in the type of firm ownership.

	(1)	(2)	(3)	(4)
	Non-SOEs	SOEs	Non-SOEs	SOEs
	InQuantity	InQuantity	InQuality	InQuality
lnEdu	0.048***	0.049***	0.055***	0.052***
	[7.25]	[7.27]	[9.15]	[8.54]
lnSize	0.366***	0.478***	0.408***	0.483***
	[26.11]	[28.52]	[31.98]	[31.76]
ROA	−0.013	0.122***	−0.021	0.109***
	[−0.31]	[3.37]	[−0.55]	[3.30]
Lev	−0.053	0.036	−0.113**	0.004
	[−0.92]	[0.52]	[−2.16]	[0.07]
Cash	−0.100	−0.147**	−0.027	−0.094 *
	[−1.15]	[−2.40]	[−0.34]	[−1.69]
lnAge	0.151***	0.025	0.141***	−0.002
	[8.29]	[0.91]	[8.47]	[−0.06]
Top1	0.271***	−0.585***	−0.016	−0.730***
	[2.73]	[−5.39]	[−0.18]	[−7.41]
Market	−0.006	0.013	−0.015	−0.005
	[−0.54]	[0.91]	[−1.38]	[−0.40]
lnGDP	0.283**	0.027	0.614***	0.170 *
	[2.35]	[0.26]	[5.61]	[1.76]
Year effect	Yes	Yes	Yes	Yes
Individual effects	Yes	Yes	Yes	Yes
_cons	−9.436***	−9.654***	−13.501***	−10.968***
	[−8.04]	[−9.54]	[−12.62]	[−11.92]
N	24635	15458	24635	15458
R-sq	0.261	0.385	0.291	0.407

* , **, and *** show statistical significance at 10%, 5%, and 1%, respectively.

However, the opposite results are found regarding the quality of innovation output, as a greater impact on the outcome is identified at non-SOEs. This might be because non-SOEs receive less support from the state, so they need to put more effort into innovative development and understand how to weigh the interests between substantive innovation and long-term enterprise development, thus effectively motivating innovation by employees. Our comparison of the impact of human capital on innovation performance at enterprises with the same type of ownership shows that SOEs pay more attention to the quantity of innovation output. In contrast, non-SOEs pay more attention to its quality. Optimizing the structure of human capital has a more substantial impact on innovation at non-SOEs than SOEs. However, technological innovation at SOEs still focuses more on quantity than quality, leaving room for improvement.

Table 7 reports the results of heterogeneity regarding compensation incentives. The logarithm of the increase in employee compensation for the current period measures compensation incentives. Innovation is a behavior that needs to be driven by motivation, and recognizing employee value and raising employee compensation is an effective way to motivate them. We calculate the median employee compensation in the full sample and then use it to divide the sample into two groups based on whether the amount of compensation is higher or lower than the median. The results for models 1 and 2 show that, regardless of the compensation level, investment in human capital can significantly positively impact innovation output. However, the positive influence of human capital on the quantity and quality of innovation output is weaker in the group with higher compensation. This might be because the senior managers at an enterprise often receive a higher salary. In contrast, it is generally the R&D staff who create real innovations at a higher quantity and quality, and they rarely earn as much as executives. Therefore, our results suggest that enterprises consider adjusting their salary structure to offer R&D personnel higher salaries and more benefits as incentives to drive innovation outputs.

**Table 7:** Heterogeneity in compensation incentives.

	(1)	(2)	(3)	(4)
	Low employee pay	Employees are paid well	Low employee pay	Employees are paid well
	InQuantity	InQuantity	InQuality	InQuality
lnEdu	0.050***	0.038***	0.049***	0.042***
	[7.21]	[5.49]	[7.75]	[6.71]
lnSize	0.309***	0.486***	0.296***	0.521***
	[18.48]	[24.41]	[19.83]	[28.70]
ROA	−0.009	0.131 *	−0.014	0.042
	[−0.27]	[1.77]	[−0.49]	[0.62]
Lev	0.085	−0.177**	−0.035	−0.218***
	[1.41]	[−2.30]	[−0.65]	[−3.10]
Cash	−0.160**	0.044	−0.119**	0.215**
	[−2.56]	[0.39]	[−2.12]	[2.10]
lnAge	0.111***	−0.000	0.140***	−0.026
	[5.56]	[−0.01]	[7.81]	[−1.10]
Top1	0.073	−0.081	0.061	−0.232**
	[0.62]	[−0.71]	[0.57]	[−2.25]
Market	−0.009	0.006	−0.009	−0.016
	[-0.70]	[0.44]	[−0.75]	[−1.34]
lnGDP	0.247**	0.054	0.480***	0.222 *
	[2.08]	[0.43]	[4.53]	[1.92]
Year effect	Yes	Yes	Yes	Yes
Individual effects	Yes	Yes	Yes	Yes
_cons	−8.051***	−10.019***	−10.129***	−12.216***
	[−6.98]	[−8.10]	[−9.82]	[−10.84]
N	19948	19948	19948	19948
R-sq	0.197	0.309	0.204	0.329

* , **, and *** show statistical significance at 10%, 5%, and 1%, respectively.

## 5. Robustness test

### 5.1. Two-stage least squares instrumental variables (2SLS-IV)

A firm’s human capital and innovation performance have a causal relationship. Increases in human capital can impact enterprise innovation in terms of productive power. However, improving enterprise innovation performance enhances the flow of capital, talents, technology, and so on, which facilitates the knowledge diffusion and talent agglomeration effects and can also lead to optimization of the structure of human capital. To mitigate the potential endogeneity problem caused by bidirectional causation between human capital and innovation performance, we construct an instrumental variable for the average sales price (IV) of commercial housing in the city where the firm is located and perform further testing with a two-stage least squares (2SLS) model.The results of the test are presented in Table 8 below.

**Table 8:** Estimation results for the instrumental variable.

	(1)	(2)
	InQuantity	InQuality
Stage 1 (l.lnEdu)	0.0000***	0.1500***
	(0.0000)	(4.1743)
Stage 2 (lnEdu)	0.2921***	0.4528***
	(0.0447)	(0.0205)
Control variables	Yes	Yes
Year effect	Yes	Yes
Individual effects	Yes	Yes
Kleibergen-Paap rk LM	1,219.813	1,219.813
	[0.0000]	[0.0000]
Kleibergen-Paap rk Wald F	1,452.412	1,452.412
	{16.38}	{16.38}
N	40,093	40,093
R-sq	0.2553	0.2853

Robust standard errors are in parentheses, the *p*-value is in square brackets, and the test critical value corresponding to the 10% significance level of the Stock-Yogo weak instrumental variable is in curly brackets.

First, the average sales price of commercial (i.e., private) housing is an important factor that affects a firm’s ability to attract talent [45]. On the one hand, housing costs directly reflect the cost of living in a city, which has a pull effect on the ability to attract workers. On the other hand, housing costs indirectly reflect the advantages and disadvantages of the environment and transportation convenience in a city, which also affect people’s choices about where to accept a position. Therefore, the average cost of housing and human capital structure is closely related.

Second, the average cost of private housing is not a direct factor that affects enterprise innovation, and, therefore, it meets the basic condition for being used as an instrumental variable (IV). In addition, the LM and F statistics from the first regression stage show that the IV is neither unidentifiable nor weak. The result for the IV is significant at the 1% level, which indicates a significant correlation between the IV and the explanatory variable in the current period, so the IV passes the correlation test. The results of the DWH test reject the null hypothesis at a significance level of 5%, and the explanatory variables pass the endogeneity test. Therefore, the selection of the IV is reasonable.

In the second stage, the coefficients for human capital are all significantly positive at the 1% level, indicating that the results are robust after endogeneity problems are controlled for. This further supports our previous findings.

### 5.2. Propensity score matching (PSM)

Variables for human capital might also have self-selection problems, which lead to estimation bias, so we use the propensity score matching (PSM) method to enhance the causal inferences. First, we divide human capital into groups based on the median number of employees with a bachelor’s degree level or above, forming an experimental group comprising those above the median and a control group comprising those below the median. Second, the same control variables mentioned earlier are used for matching, and enterprise innovation performance is used as the result variable. Finally, we use a Logit model to perform 1:1 nearest-neighbor matching, radius matching with a radius of less than 0.01, and quadratic kernel matching, respectively, to estimate the propensity scores of each group, as shown in Table 9. The experimental and control groups both have a value in the range (0,1), and a very small number of samples are excluded during the matching process, which satisfies the hypothesis of common support. After matching, the individual characteristics of the experimental and control groups are significantly reduced, and the model passes the balancing test.

**Table 9:** Propensity score matching.

Matching method	Result variables	Experimental group	Control group	ATT	Standard error	T value
Neighbor matching	InQuantity	2.6962	1.9579	0.7382	0.0341	21.64
	InQuality	2.5379	1.6087	0.9291	0.0297	31.27
Radius matching	InQuantity	2.6320	1.7642	0.5376	0.0573	7.65
	InQuality	2.5033	1.6533	0.7637	0.0434	8.73
Nuclear matching	InQuantity	2.6549	1.5258	0.3635	0.0535	9.63
	InQuality	2.4328	1.4433	0.2526	0.0399	10.54

Table 10 shows the results from the ordinary least squares (OLS) regression, in which the explanatory variable is the group variable for human capital, and the explained variable is firm innovation performance. The PSM results show that the result variable is either InQuantity or InQuality, regardless of whether the estimation uses nearest-neighbor, kernel, or radius matching. The average treatment effect of the treated (ATT) of human capital is consistent with the estimation results of the OLS regression, which indicates that the structure of human capital has a significantly positive impact on the innovation performance of enterprises, which further confirms the robustness of our findings.

**Table 10:** OLS regression results.

Variables	(1)	(2)	(3)	(4)
	InQuantity	InQuantity	InQuality	InQuality
dum_lnEdu	1.126***	0.428***	1.274***	0.654***
	[69.62]	[24.02]	[87.02]	[40.86]
lnSize		0.496***		0.504***
		[64.51]		[73.01]
ROA		0.132***		0.088**
		[2.97]		[2.22]
Lev		0.035		−0.565***
		[0.82]		[−14.65]
Cash		−0.302***		−0.384***
		[−4.00]		[−5.65]
lnAge		−0.147***		−0.145***
		[−16.29]		[−17.92]
Top1		−0.289***		−0.885***
		[−5.56]		[−18.94]
Market		−0.059***		−0.010 *
		[−9.60]		[−1.75]
lnGDP		0.470***		0.350***
		[40.69]		[33.73]
_cons	1.589***	−13.112***	1.285***	−12.291***
	[138.76]	[−74.07]	[124.03]	[−77.27]
N	40093	40093	40093	40093
R-sq	0.108	0.244	0.159	0.296

* , **, and *** show statistical significance at 10%, 5%, and 1%, respectively.

## 6. Conclusion

This study makes some theoretical and practical contributions to the literature. First, our results confirm a positive relationship between the optimization of the human capital structure and firm innovation performance based on our data analysis. Unlike prior literature, this paper divides corporate innovation performance into substantive innovation and strategic innovation, which enhances the measurement of corporate innovation performance to some extent. Adding this distinction helps companies determine whether they are receiving a substantial return on their investment in innovation. Similarly, from the perspective of external investors and stakeholders, it can demonstrate whether an enterprise has the potential for innovative development through investment rather than support from subsidies to make future investment decisions or will only produce marginal innovation output. In addition, our analysis of the mediating effect confirms that optimization of the structure of a firm’s human capital can affect innovation performance through technology intensity and R&D investment and receiving government subsidies and shows a partial mediating effect. Thus, our study offers theoretical references on how human capital can raise firm innovation performance.

The heterogeneity analysis reveals that, after we divide the sample enterprises into SOEs and non-SOEs, optimization of the human capital structure has a more substantial effect on innovation at non-SOEs. In contrast, technological innovation is heavy on quantity and light on quality at SOEs. Although the difference in the results between the two is small because our sample is limited to Chinese A-share listed enterprises, the gap between the two groups of enterprises might be larger if we considered a wider range of Chinese enterprises. This finding can lead to future research to explore whether SOEs differ significantly from non-SOEs in relevant national policies, government subsidies, and talent attraction, as well as their utilization efficiency of input costs, yielding different innovation performance results. In addition, our examination of whether employee compensation impacts enterprise innovation performance shows that increasing compensation can transform innovation quantity into quality. However, contrary to the conventional wisdom, we find that the R&D staff are more likely to produce substantive innovation than high-level managers. This also offers new ways of thinking about how to increase substantive innovation by enterprises in the future, for example, by adjusting the salary structure at firms for managers versus ordinary employees to obtain greater rewards from investment in innovation. Our results may hint at the reasons for poor innovation performance by some enterprises and suggest ways to help enterprises optimize their innovation performance in the future.

Our findings lead to some management implications. For enterprises, optimizing human capital structure plays a significant role in driving innovation performance. Enterprises should pay attention to recruiting and cultivating those with high professional and digital skills to attract the human capital necessary for accelerating firm innovation.

First, enterprises should fully optimize their human resource management and establish mechanisms for training digital talent and enhancing professional skills internally and in cooperation with universities and research institutions to raise incumbent employees’ digital literacy and creativity.

Second, enterprises should seek to hire digital innovation talents who are highly knowledgeable and capable of operating and mastering digital technology to adapt to enterprises’ innovative production processes and management modes. This requires recruiting new employees based on more careful consideration, including but not limited to direct education, skill certificates, and other proof, and attention should be paid to the inspection of professional skills. Corporate recruiters should receive regular training to enhance the professionalism of a firm’s employees and to raise efficiency in recruitment.

Third, the structure of human capital and demand for enterprise innovation and the structure of human capital and demand for employment need to be more closely aligned. Human capital plays a role in innovation performance in part when the number of highly skilled employees who increase the technological intensity increases. However, highly qualified personnel are concentrated at China’s higher education or research institutions. This is mainly due to the significant difference in orientation between human goals and corporate goals, leading to human capital scarcity and, at the same time, organizational redundancy [46,47]. Therefore, enterprises should consider raising employee compensation and obtaining greater subsidies to attract highly skilled employees and optimize human capital structure.

Finally, local governments should vigorously pursue strategies to attract highly skilled employees to enterprises in their jurisdiction and drive innovative development by enterprises, especially non-SOEs. Enterprises face financial pressure, have a limited ability to attract high-quality talent, are closer to the market, and have a higher demand for innovation; helping them recruit talent would help raise the overall level of innovation in the country. This can be done if local governments design relevant policies and provide financial subsidies and other incentives, such as strengthening the protection of employees’ rights and interests and offering training to enhance employees’ skills. In addition, the structure of government subsidies should be optimized to promote balanced development across various types of enterprises. Those for non-SOEs and startups are relatively small among the existing government subsidies, even though they are sources of innovation closer to the market and need help that external resources could provide.

Because this paper shows that, in China, the pursuit of innovation performance through enhancing human capital relies in part on receiving larger government subsidies, reconsideration of the structure of government subsidies for all industries and all types of firms is sorely needed to help developing firms achieve innovation and create an environment that encourages innovation. This requires the government to create better methods for determining which enterprises should receive subsidies and other incentives to support performance based on their high innovation ability. At the same time, implementing these policies should avoid assuming that one size fits all, and a regular assessment should be performed to evaluate whether enterprises achieve long-term and sustainable innovation effects. This would also contribute to creating an environment that truly attains innovation.
